# Annual Observation of Changes in the Angle of Trunk Rotation. Trunk Asymmetry Predictors. A Study from a Scoliosis Screening in School Adolescents

**DOI:** 10.3390/ijerph17061899

**Published:** 2020-03-14

**Authors:** Katarzyna Adamczewska, Marzena Wiernicka, Ewa Kamińska, Joanna Małecka, Agata Dąbrowska, Ewa Malchrowicz-Mośko

**Affiliations:** 1 Faculty of Health Sciences, Poznan University of Physical Education, 61-871 Poznan, Poland; wiernicka@awf.poznan.pl (M.W.); kaminska@awf.poznan.pl (E.K.); malecka@awf.poznan.pl (J.M.); 2 Faculty of Sport Sciences, Poznan University of Physical Education, 61-871 Poznan, Poland; zagata.pl@gmail.com (A.D.); malchrowicz@awf.poznan.pl (E.M.-M.)

**Keywords:** angle of trunk rotation, idiopathic scoliosis, school scoliosis screening, trunk asymmetry determinants, children

## Abstract

Adolescent forms of idiopathic scoliosis are commonly encountered deformities of the thoracic and lumbar spine. They affect a significant number of adolescents, yet their cause is still unknown. The presented research is a cross-sectional analysis of 3933 volunteers (2131 girls and 1802 boys). The participants were primary school students aged 9 to 13 years old. This study determined a relationship between predictors such as: body mass, body height and body mass index (BMI) (independent variables) and angle of trunk rotation (ATR) value (dependent variable). Moreover, a stepwise multiple regression with backward selection was conducted to determine to what extent the dependent variable is explained by body mass, body height and BMI. In the group of 11,12,13-year-old girls, the analyzed results of multiple stepwise regression were statistically significant. Among the all studied predictors, it has been shown that body mass in the 11-year-old girls and body height in 12- and 13-year-old girls are major correlates of a 1-year ATR increase in proximal and main thoracic spine levels.

## 1. Introduction

Adolescent idiopathic scoliosis (AIS) curves are a complex of three-dimensional deformities that require adequate correction in all three dimensions, coronal, sagittal and axial planes [1]. The adolescent forms of IS are commonly encountered deformities of the lumbar and thoracic spine. It affects 2%–3% of adolescents. Researchers have focused on genetic factors, metabolic and hormonal disorders [2], growth asymmetry with mechanical and connective tissue abnormalities, asymmetrical and high loads associated with wearing school backpacks, and the asymmetry of load distribution in the lower limbs [3]. At the early stage of IS, the symptoms are not so visible [4]. Children and adolescents with scoliosis might look thinner and taller than normal [5]. An ectomorphic body shape (thin), especially in girls, is considered to be a risk factor for idiopathic scoliosis [6]. In addition, the anthropometric character of scoliosis patients is thought to be related to the pathophysiology of scoliosis, namely the influence of leptin as well as somatic and authonomic nerve disharmony [7]. According to Negrini et al. (2007), it was observed that postural response to the asymmetrical load (8 kg) was retropositioning (4 mm). The patients showed an elevation (2,5 cm) of the loaded shoulder together with a lateral deviation of the trunk (3 cm) away from the load [8]. They also confirmed that loading the spine of a 12-year-old student, symmetrically or otherwise, always prompts postural variation [9]. Nery et al. (2015) confirmed that carrying a schoolbag weighing 10% of one’s body mass or more, could generate back muscle tone disturbances leading to scoliosis [10]. Similar findings have been presented in other scientific reports [11]. The implementation of the scoliosis school screening program is inextricably bonded to non-operative idiopathic scoliosis treatment [12]. It is certain that pain may arise in any patient, regardless of the curve size and location. What is important is the back pain in youngsters is correlated with back pain in their adulthood [13]. School screening programs, if conducted at all, should aim for low false-positive rates. High diagnostic error rates cause concern among adolescents and their parents and force them to have unnecessary radiographs and specialty consultations [14].

The implication of the scoliometer examination with the combination of the Adams Forward Bending Test was used to assess trunk asymmetry. This tool was chosen because of its good specificity, sensitivity and predictive capability (correlation with the radiographic analysis r = 0.7 with *p* < 0.05) [15].

The aim of the study was to (1) detect the children who had an increase in ATR values in at least at one of the spine levels examined in a one-year follow-up, (2) present the differences between the angle of trunk rotation (ATR) values in annual observation, (3) determine the relationship between body mass, body height, BMI and angle of trunk rotation value in school children.

## 2. Materials and Methods

In the presented research, a cross-sectional and observational method of study was used. The examination project was approved by the University of Medical Sciences Bioethics Committee under process number 892/12. 

All the evaluators received appropriate training before starting the trunk asymmetry measurements. The methodological guidelines were strictly followed. Parents and legal guardians of all volunteers signed an informed consent form prior to participation.

### 2.1. Characteristics of Population

The research covered 65 schools in the metropolitan of Poznan in Poland. The study analysed 6850 volunteers (*n* = 6850, 3440 girls and 3410 boys). Only the participants, whose mean ATR value increased during the second trial, were included to create an asymmetry determinants regression model. A total of 3933 volunteers (2131 girls and 1802 boys) were selected from an initial database for further research. All the respondents met the inclusion criteria. The participants were between 9 and 13 years old and took part in regular school physical activities in the last year. In addition, all the volunteers included in the study had an annual increase in the angle of trunk rotation value of at least one measurement level of the spine. The exclusion criteria were: previous spine or lower limb surgery, neurological disorders, the difference in lower limbs length smaller than 2,5 cm.

### 2.2. Research Procedure

The anthropometric data and the angle of trunk rotation value were assessed two times in the study. After the second trial, between the initial and final values, the delta body mass, body height and ATR were calculated. Each duration of the trial lasted 1 month. The time between the first and the second examination took 10 months. Measurements were made by forty-five qualified therapists.

The patients’ anthropometric characteristics were examined in Frankfort’s position without wearing shoes or footwear. Weight and height were measured, with the subject standing, to the nearest 0.1 kg and 0,1 cm, respectively, using a scale which was also equipped with a stadiometer (HM200P Port Stad Portable). BMI was calculated in the standard way, using the formula: weight (kg) divided by squared height (m^2^) [16].

During the scoliosis examination, which was performed using a Bunnell scoliometer, the boys were evaluated shirtless. Girls wore a customized backless t-shirt to allow full view of their back. Female participants had their hair tied up. The screening test was easy to carry out, non-invasive and safe for the respondents. The Scoliometer examination reveals good repeatability and reproducibility [17]. In addition, this tool is a simple device that can be used to measure axial rotation of the spine. The degree of axial rotation has a strong correlation with the Cobb angle, with a sensitivity of 87% [18]. For a cut-off value of the ATR equal to or greater than 7°, the scoliometer examination is characterized by a high sensitivity (83,3%) and high specificity (86,8%) [19]. Bonagamba et al. (2010) suggested that it can be inferred that the major sources of variability of the measurement performed with the scoliometer come from the process of positioning, and the palpation and determination of the spinous process, not just the record of the measures of axial rotation with the device [20].

Through palpation, the spinous processes T_1_ to L_5_ and the posterior iliac superior spine were located and marked using easy-blend and anti-allergic markers. This procedure provides information of what is happening with the spinous processes in the frontal and axial plane. For clinical reasons, it is very helpful for therapists to make skin markings. The measurement was performed in the standing position during forward bending of the trunk (Adam’s forward bending test). This test is a direct visual observation to see if there are any abnormalities in the back, such as a hump or asymmetry of the back [21]. The ATR measures were done at three levels of the spine. Lenke et al. (2003) defined the proximal thoracic spine from T_2_–T_5_, the main from T_6_–T_9_, and thoraco-lumbar from T_10_–_L2._) [22].

The patients bent their trunk forward until it was parallel to the ground, keeping the palms of their hands together. The volunteers’ feet were kept approximately perpendicular with the iliac junction line. The examination took place in school, in separate bright rooms with adequate thermal conditions. If the participants had an ATR > 3° degrees, they were strongly suspected for trunk asymmetry. Therefore, these volunteers were referred for follow-up examinations within three months.

All the statistical analysis was performed using StatSoft, Inc. (2011). STATISTICA (data analysis software system), version 10 (University of Physical Education, Poznan, Poland). The descriptive statistics were calculated for baseline demographics, and scoliometer’s results for the entire sample. The stepwise multiple regression with backward selection was conducted to test the influence of independent variables on the variance in angle of trunk rotation value. Only the independent factors that correlated significantly with the dependent variables during the multiple regression analysis were included in the model. The critical level of significance was set at α = 0.05.

## 3. Results

### 3.1. Demographics

In the Table 1, the demographics of all studied individuals were presented.

### 3.2. The Multiple Regression Model for Predictors Determining the Mean Values of ATR Increase

The relationship between ∆ ATR, and the body mass, body height, and BMI introduced in the stepwise multiple regression analysis was not statistically significant in girls aged 9 and 10 years.

For the asymmetry predictors on the proximal thoracic spine in a group of girls aged 11 years, the results of the stepwise multiple regression analysis indicated that there were positive relations between body mass and ATR value, and body height and ATR value (Table 2).

The relationship between the body mass, body height and BMI, introduced in the first step in a regression model, was not statistically significant. Body mass and BMI explained, in total, about 21% of the variance in trunk asymmetry (*p* < 0.05), while body mass, introduced into the third step, explained an additional ~20% of variance in ∆ ATR. Higher body mass values coincided with a greater angle of trunk rotation values on the proximal thoracic spine. The relationship between body mass, body height and BMI, and its influence on ATR increase on the main thoracic and thoraco-lumbar spine, were not statistically significant.

In the case of the asymmetry predictors on the proximal and main thoracic level of the spine in a group of girls aged 12 years, the results of the stepwise multiple regression analysis indicated that there were positive relations between body mass and ATR value, body height and ATR value, and BMI and ATR value (Table 3).

The results presented in Table 3 have shown that relations among all the predictors are positive. On the proximal thoracic spine, body mass, body height and BMI altogether accounted for about 37% of variance in ∆ ATR (*p* < 0.05), with body mass as the strongest predictor. On the main thoracic spine, there was a strong relation between body mass, body height and BMI predictors with ∆ ATR (*p* < 0.001). The regression model explained 62% of the variance. In this case, the strongest asymmetry predictor was body height (*p* < 0.001). Due to the high level of statistical significance of the results, the mathematical equations of the second and third steps have been suspended. The relationship between body mass, body height and BMI and its influence on ATR increase on the thoraco-lumbar spine were not statistically significant.

In the case of the increase in ATR value on the proximal thoracic spine in girls aged 13 years, the results of the stepwise multiple regression analysis indicated that there were positive relations between body mass and ATR value, and body height and ATR value (Table 4).

In the first step, the full regression equation was statistically significant (*p* < 0.05). BMI was excluded from the regression model in the second step. As a result, the body mass and body height together accounted for 39% of variance (*p* < 0.001). In that case, the body height was proved to be the strongest predictor. Body height alone explained about 21% of the variance (*p* < 0.05) in the third step of the stepwise multiple regression analysis. The relationship between body mass, body height and BMI, and its influence on the ATR increase in the main thoracic and thoraco-lumbar spine, was not statistically significant.

In the group of boys aged 9 to 13 years, at all levels of the spine, the results of the stepwise multiple regression analysis were not statistically significant.

## 4. Discussion

The early detection of curves has been facilitated by school-based screening, but has resulted in a need to understand the natural history and reliable prediction of curve progression to decide on the appropriate treatment and timing of intervention [23]. According to the presented article, the results of the stepwise multiple regression analysis indicated relationships between body mass and ATR value, body height and ATR value, and BMI and ATR value. It has been confirmed that primary scoliosis spine deformities appear most often in the proximal and the main thoracic spine, and also during the most intense posturogenesis. Usually, in girls, this is from 11 to 13 years old. The results of this study have shown that the first changes in spine curves appeared in a group of 11-year-old girls. These changes are primarily associated with the increase in body mass, while, in 12- and 13-year-old girls, the predictor that most affects the increase in angle of trunk rotation is the body height. This age is characterized by a greater increase in body height, which causes the spine lability and less resistant to the deformation in the axial plane. This seems to be very important in the context of developing an appropriate therapy and preventing the progression of trunk asymmetry.

The regression model has revealed a different relationship among individual predictors in all age and sex groups. The latest research has confirmed that more girls than boys have an angle of trunk rotation of more than 5 degrees, especially between the 11- and 13-year olds [2]. Coelho et al. (2013), in their study, showed that it is possible to detect 100% curves greater than 20° Cobb in patients using 5° of ATR as a criterion for evaluation [15]. Perhaps that is why regression models are more relevant for girls at all levels of measurement.

Some studies have confirmed that the reduced body weight, especially in a group of girls, strongly predisposes the occurrence of scoliosis. The analysis of results by Brazolino et al. (2015) has shown that adolescents with scoliosis had a lower body mass than normal [24]. In the presented research, an attempt was made to find predictors that will affect the increase in trunk asymmetry. The study results have shown that the regression model was found to have high confidence, especially in girls. The results have confirmed that the girls in the annual observation who had trunk asymmetry, in order to decrease the ATR value, cannot significantly increase their body weight. The weight gain in patients with scoliosis may have a negative impact, leading to increased deformities of the spine. Goodbody et al. (2017) reported that overweight and obese patients with AIS showed larger curve magnitudes (24- and 25-degree major curves, respectively) compared with normal-weight patients (18-degree major curve) [25]. Obese children had larger major curves at the time of presentation compared with their normal-weight counterparts [26].

Body height was another factor analyzed in the regression models. It may play a role in the etiology of AIS, but it certainly affects the pathology. Ylikoski (2003) showed that adolescents with scoliosis are taller than other adolescents in the same age group. The results have confirmed that differences in body height between normal and scoliosis students were much greater [27]. It also was found by Ylikoski (2005) that a growth velocity of more than 2 cm per year is associated with curve progression [28]. It is believed that the increased prevalence of scoliosis in girls compared to boys is justified by the fact that girls tend to grow more than boys from ages 11 to 13 [29]. This period of growth spurt contributes to the emergence of postural changes in childhood and adolescence [30]. In the presented study, the analysis of the results has shown that both regression models obtained in a group of girls and boys indicate an increase in body height that effects the increase in trunk asymmetry. A similar regression model applies for the BMI value. BMI is an expression of the ratio of body weight to height, and it will be considered a factor determining the increase in angle of trunk rotation. Kyoungkyu et al. (2018) indicated that school students aged 10 to 14 years in the underweight and severely underweight groups had a significantly higher risk of developing scoliosis, having risk levels that were 1.43 and 1.45 times higher, respectively [31].

Contrary to other studies, the presented results can determine the predictors that affect 1-year ATR increase. Primarily taking regression models into account, the predictors should be considered during the planning and implementation of therapy and exercises in patients with scoliosis.

Performing scoliosis screening programs to exclude children with suspected scoliosis is very important. The studies on AIS prevalence have demonstrated that the number of patients who suffer pain vary from 27% to 59% [32]. In addition, it may be associated with other conditions such as restricted ventilation, respiratory muscle weakness, reduced quality of life [33], and even psychological problems [34].

### Strengths and Limitations

This study is limited by some factors. The presented study was conducted in five age groups. Therefore, the data cannot be extrapolated to other age ranges. The other limitation is that the scoliometer values were measured by forty-five therapists, therefore, interobserver and intraobserver reliability was not assessed. Despite these limitations, the results provide valuable knowledge from the stepwise multiple regression analysis that indicated positive relations between body mass and ATR value, body height and ATR value, and BMI and ATR value. In the future, advanced research may include a wider group of predictors which also will have an influence on the dependent variable.

## 5. Conclusions

In summary, it has been shown that body mass in 11-year-old girls and body height in 12- and 13-year-old girls are major correlates of a 1-year ATR increase in the proximal and main thoracic spine levels. In case of 11-year-old girls, the monitoring of body weight seems to be very important to detect abnormal trunk asymmetry. In case of excessive body weight gain, dietary control and an increase in physical activity might be proposed to prevent excessive ATR increase. On the other hand, in girls aged from 12 to 13 years, the monitoring of body height should be recommended to prevent too intense an increase in ATR. In case of rapid body height increase, appropriate soft brace and physical treatment might be recommended to limit the progression of ATR and development of spinal deformities.

## Figures and Tables

**Table 1:** Basic characteristics of participants (*n* = 3933) presented in annual increases described as the mean, minimum, maximum and standard deviation.

**Variable**	***n***	**Mean**	**Min**	**Max**	**SD**	***n***	**Mean**	**Min**	**Max**	**SD**
	**Girls (9 Years Old)**					**Boys (9 Years Old)**				
∆ Weight	256	2.6	−8.4	8.7	1.9	202	2.9	−6.3	14.8	2.2
∆ Height	256	5.4	2.0	15.0	1.3	202	5.7	2.5	15.0	1.7
∆ BMI	256	0.2	−6.8	3.3	1.1	202	0.2	−5.7	5.6	1.2
∆ ATR 1	158	1.7	1.0	5.0	0.9	97	1.7	1.0	8.0	1.1
∆ ATR 2	169	1.9	1.0	8.0	1.2	139	1.9	1.0	6.0	1.1
∆ ATR 3	156	1.8	1.0	6.0	0.9	104	1.7	1.0	4.0	0.8
	**Girls (10 Years Old)**					**Boys (10 Years Old)**				
∆ Weight	535	3.1	−6.8	12.0	1.9	488	3.2	−4.5	26.0	2.2
∆ Height	535	5.3	1.9	14.0	1.3	488	5.3	1.5	29.0	1.7
∆ BMI	535	0.3	−5.8	3.6	0.9	488	0.4	−3.4	5.0	0.9
∆ ATR 1	331	1.7	1.0	12.0	1.2	264	1.8	1.0	7.0	1.0
∆ ATR 2	354	1.9	1.0	9.0	1.3	339	1.9	1.0	7.0	1.2
∆ ATR 3	327	1.8	1.0	10.0	1.2	273	1.8	1.0	10.0	1.2
	**Girls (11 Years Old)**					**Boys (11 Years Old)**				
∆ Weight	534	3.4	−2.8	14.9	2.2	451	3.5	−4.2	16.7	2.2
∆ Height	534	5.4	0.8	15.5	1.7	451	5.1	1.0	11.0	1.4
∆ BMI	534	0.4	−3.1	6.5	1.0	451	0.4	−5.2	7.7	1.0
∆ ATR 1	316	1.7	1.0	7.0	1.0	245	1.7	1.0	7.0	1.0
∆ ATR 2	335	1.9	1.0	18.0	1.4	300	1.9	1.0	6.0	1.1
∆ ATR 3	331	1.7	1.0	5.0	1.0	259	1.7	1.0	10.0	1.1
	**Girls (12 Years Old)**					**Boys (12 Years Old)**				
∆ Weight	499	3.9	−16.7	14.6	2.7	390	3.2	−8.8	16.8	2.4
∆ Height	499	5.9	1.5	19.0	1.9	390	4.9	1.5	15.5	1.5
∆ BMI	499	0.3	−9.0	5.0	1.2	390	0.3	−5.8	6.0	1.0
∆ ATR 1	306	1.8	1.0	15.0	1.4	207	1.8	1.0	7.0	1.0
∆ ATR 2	311	2.0	1.0	9.0	1.3	238	1.8	1.0	7.0	1.1
∆ ATR 3	291	1.8	1.0	7.0	1.1	232	1.8	1.0	7.0	1.0
	**Girls (13 Years Old)**					**Boys (13 Years Old)**				
∆ Weight	307	4.7	−8.2	13.3	2.9	271	3.9	−3.0	14.2	2.6
∆ Height	307	6.0	1.5	17.5	1.8	271	5.4	1.5	15.0	1.9
∆ BMI	307	0.6	−4.6	4.1	1.2	271	0.4	−2.3	3.8	1.0
∆ ATR 1	175	1.9	1.0	7.0	1.3	156	1.8	1.0	7.0	1.1
∆ ATR 2	210	1.9	1.0	8.0	1.3	175	2.0	1.0	6.0	1.1
∆ ATR 3	189	1.7	1.0	6.0	0.9	163	1.7	1.0	6.0	0.9

*n*—the number of children in whom the mean increase in angle of trunk rotation (ATR) value was noted in the second trial of the examination, SD—standard deviation, ∆—delta, a difference between the final and initial values, ∆ ATR 1—the delta of mean increase in ATR value at the proximal thoracic (T_2_–T_5_) in the second trail of the examination, ∆ ATR 2—the delta of mean increase in ATR value at the main thoracic (T_6_–T_9_) in the second trail of the examination, ∆ ATR 3—the delta of mean increase in ATR value at the lumbar (T_10_–L_2_) in the second trail of the examination.

**Table 2:** The regression analysis predicting the mean of ATR value in girls aged 11 years (*n* = 534).

Level	Variable		*p*	F, *p*	R^2^	Change R^2^
Proximal Thoracic	Step 1	Body mass	0.412	2.25, 0.106	0.206	0.115
		Body height	0.866			
		BMI	0.911			
	Step 2	Body mass	**0.065**	**3.50, 0.045**	**0.206**	0.147
		Body height	**0.835**			
		BMI	–			
	Step 3	Body mass	**0.012**	**7.19, 0.012**	**0.204**	0.176
		Body height	–			
		BMI	–			

**Table 3:** The regression analysis predicting the mean of ATR value in girls aged 12 years (*n* = 499).

Level	Variable		*p*	F, *p*	R^2^	Change R^2^
Proximal Thoracic	Step 1	**Body mass**	**0.005**	**3.79, 0.0276**	0.374	0.275
		Body height	**0.016**			
		BMI	**0.032**			
Main Thoracic	Step 1	Body mass	**<0.001**	**11.4, <0.001**	0.620	**0.565**
		**Body height**	**<0.001**			
		BMI	**<0.001**			

**Table 4:** The regression analysis predicting the mean of ATR value in girls aged 13 years (*n* = 307).

Level	Variable		*p*	F, *p*	R^2^	Change R^2^
Proximal Thoracic	Step 1	Body mass	0.192	4.00, **0.0266**	0.428	0.321
		Body height	0.126			
		BMI	0.332			
	Step 2	Body mass	0.035	**5.49, 0.014**	0.393	0.321
		**Body height**	**0.004**			
		BMI	–			
	Step 3	Body mass	–	4.67, **0.044**	0.206	0.162
		Body height	0.044			
		BMI	–			
